# Therapeutic effect and mechanism of Daikenchuto in a model of methotrexate-induced acute small intestinal mucositis

**DOI:** 10.1371/journal.pone.0283626

**Published:** 2023-03-30

**Authors:** Peilin Li, Yusuke Inoue, Daisuke Miyamoto, Toshiyuki Adachi, Satomi Okada, Tomohiko Adachi, Akihiko Soyama, Masaaki Hidaka, Kengo Kanetaka, Shinichiro Ito, Daichi Sadatomi, Sachiko Mogami, Naoki Fujitsuka, Weili Gu, Susumu Eguchi

**Affiliations:** 1 Department of Surgery, Nagasaki University Graduate School of Biomedical Sciences, Nagasaki, Japan; 2 Department of Surgery, Guangzhou First People’s Hospital, School of Medicine, South China University of Technology, Guangzhou, Guangdong, China; 3 Tsumura Kampo Research Laboratories, 2 Tsumura Advanced Technology Research, Tsumura & Co., Ibaraki, Japan; University of Maryland Baltimore, UNITED STATES

## Abstract

**Background:**

Daikenchuto (DKT) has positive therapeutic effects on improving various gastrointestinal disorders. The present study investigated whether or not DKT has a potential therapeutic effect on chemotherapy-induced acute small intestinal mucositis (CIM) in a rat model.

**Methods:**

Intraperitoneal injection of 10 mg/kg methotrexate (MTX) every 3 days for a total of 3 doses was used for induction of CIM in a rat model. The MTX and DKT-MTX groups were injected with MTX as above from the first day, and the DKT-MTX and DKT groups were administered 2.7% DKT via the diet at the same time. The rats were euthanized on day 15.

**Results:**

The DKT-MTX group showed an improvement in the body weight and conditions of gastrointestinal disorders as well as increased levels of diamine oxidase in plasma and in the small intestinal villi. The pathology results showed that small intestinal mucosal injury in the DKT-MTX group was less severe than that in the MTX group. Immunohistochemistry for myeloperoxidase and malondialdehyde and quantitative real-time polymerase chain reaction (RT-qPCR) for TGF-β1 and HIF-1α showed that DKT attenuated peroxidative damage. The crypts in the DKT-MTX group contained more Ki-67-positive cells than MTX group. The zonula occluden-1 and claudin-3 results showed that DKT promoted repair of the mucosal barrier. RT-qPCR for the amino acid transporters EAAT3 and BO+AT also confirmed that DKT promoted mucosal repair and thus promoted nutrient absorption.

**Conclusion:**

DKT protected against MTX-induced CIM in a rat model by reducing inflammation, stimulating cell proliferation, and stabilizing the mucosal barrier.

## Introduction

Intestinal mucositis (IM) is a common and debilitating side effect of chemotherapy that manifests due to the inability of chemotherapy drugs to differentiate between normal and tumor cells, with the intestinal epithelial cells rapidly proliferating cells and often becoming the target of attack during chemotherapy treatments [1]. Chemotherapy-induced IM (CIM) occurs in as many as 25%-75% of cancer patients receiving different chemotherapy, leading to diarrhea, a decreased quality of life, treatment intolerance resulting in discontinuation, and even death [2]. Due to the complex and diverse clinical symptoms of CIM and the importance of the reducing this toxic complication of chemotherapy, developing new ways to alleviate or prevent CIM is important.

Methotrexate (MTX), a structural analogue of folic acid, is one of the most widely used therapeutic agents for the treatment of the tumors, malignant hematological disorders, and autoimmune diseases [3]. MTX causes inhibition of growth and repair activities of epithelium and mucosa where the dividing and proliferating activities are increased, inducing CIM, which is the main reason for limiting further use of this drug or prompting its use [4].

Daikenchuto (DKT) is a traditional Japanese medicine (Kampo) originally described in a Chinese classic article and independently developed in Japan. It is a mixture of extract powders from dried Japanese pepper, processed ginger, ginseng radix, and malt sugar powder and is reported to have the effects of improving gastrointestinal motility, activating anti-inflammatory, increasing intestinal blood flow, and altering the intestinal microbiome [5,6]. The main mechanism underlying the DKT-mediated contraction and improvement of gastrointestinal motility is modulation of intestinal contraction and relaxation via the release of acetylcholine, nitric oxide, releasing of acetylcholine from cholinergic nerves stimulated by 5-HT3R and 5-HT4R and other excitatory neurotransmitters [7,8]. The anti-inflammatory effect of DKT are attributed to the fact that DKT manages the downregulation of cyclooxygenase 2 (COX-2), the upregulation of endogenous adrenomedullin (ADM), and the suppression of eosinophil infiltration [9]. The regulation of intestinal blood flow by DKT is achieved by stimulating epithelial transient receptor potential ankyrin 1 (TRPA1) to induce endogenous ADM release [10]. Recent studies have suggested that DKT can alter the gut microbiota, thereby improving long-lasting dysbiosis and gastrointestinal dysfunction after bowel or liver surgery [11].

In addition to these effects, Wada et al. also demonstrated that DKT enhanced anastomotic healing via an anti-inflammatory effect and increased blood flow after intestinal surgery in rats [12]. DKT has been widely used clinically in patients with gastrointestinal symptoms, such as postoperative intestinal obstruction, inflammatory bowel disease, abdominal pain, and pain accompanied by abdominal flatulence [13–15]. DKT improves gastrointestinal motility disorders and reduces serum C-reactive protein levels in patients with grade B liver injury after hepatectomy and is an effective treatment after hepatectomy for hepatocellular carcinoma [16]. A previous report showed that DKT can suppress the adverse effects associated with irinotecan hydrochloride, an anticancer agent with debilitating side effect of severe diarrhea, and improve the function of tight junction proteins, including zonula occluden-1 (ZO-1), occludin and claudin-4 [17].

The lack of relevant medication for CIM is usually only alleviated by reducing the dose of chemotherapy drugs or stopping chemotherapy, and there is a lack of relevant drugs for complementary or supplementary treatment. Based on existing experiments and recent studies on DKT, we hypothesized that DKT could improve CIM through the above mechanisms. Therefore, the present study investigated whether or not DKT could improve CIM and promote recovery from CIM.

## Methods

### Animal

Male *Sprague-Dawley rats* (6 weeks, 160–190 g; CLEA Japan Inc., Tokyo, Japan) were used in this study. They were bred and housed at the rat facility in standard rat cages exposing to 12‐h light‐dark cycles and allowed *ad libitum* access to water and rat chow.

All animal experiments were conducted according to protocols approved by the institutional animal care committee of Nagasaki University and all methods were performed in accordance with the relevant guidelines and regulations of Nagasaki University. The study was reported in accordance with ARRIVE guidelines.

### Experimental protocol

All animals were divided randomly into 4 groups: the control group (Col, n = 5), MTX-induced model group (MTX, n = 5), DKT treatment group (DKT-MTX, n = 5), and DKT-only group (DKT, n = 5). In the MTX and DKT-MTX groups, MTX was administered (10 mg/kg every 3 days, 3 times total) with intraperitoneal injection. In the MTX-DKT and DKT groups, DKT at 2.7% of the total mass (Tsumura & Co., Tokyo, Japan) mixed in the feed (CE-2 feed; CLEA Japan Inc.) was administered orally from the beginning of the administration of MTX, while the other two groups received non-DKT CE-2 feed. In the control group, the rats were intraperitoneally injected with the same volume of normal saline at the same time.

All rats had their body weight measured every three days. All surviving rats were euthanized by cutting the vena cava to induce exsanguination after abdominal collection of all of the small intestinal tissues under deep isoflurane (Wako Pure Chemical, Osaka, Japan) respiratory anesthesia (All-in-one Anesthetizer, Muromachi Kikai CO. LTD, Japan).

### Intestinal histology

The rats were sacrificed under anesthesia at day 15, and the small intestinal tissues were collected immediately. The rat small intestinal tissue was fixed with 4% paraformaldehyde phosphate-buffered solution (PBS; Wako Pure Chemical, Osaka, Japan) for 3 days. Fixed tissues were embedded in paraffin, cut into 5-μm sections, and deparaffinized for standard histological staining with hematoxylin and eosin (HE). HE sections were evaluated blindly for intestinal inflammation, which comprised crypt length, architecture and abscesses, loss of goblet cells, tissue damage, and infiltration of leukocytes and neutrophils.

For immunohistochemistry staining, tissue sections of the small intestines were stained for claudin-3, ZO-1, neutrophil myeloperoxidase (MPO), malondialdehyde (MDA), Ki-67 and diamine oxidase (DAO) (The antibody information were attached to S1 Table). The percentage of total area of the small intestinal sections was measured using image J Software analysis at least 10 positions.

### Quantitative real-time polymerase chain reaction (qRT-PCR)

Tissue samples were acquired at defined time point for mRNA extraction using spin columns according to the manufacturer’s instructions (NucleoSpin RNA II; Macherey-Nagel, Duren, Germany). cDNA was synthesized from total RNA using a high-capacity cDNA reverse transcription kit (Applied Biosystems, Tokyo, Japan). In brief, PCR amplification was performed followed by Applied Biosystems (Taq-man was attached to S2 Table). The gene expression was normalized to that of GAPDH (control intestinal tissue was set as 1.0), and the mRNA expression was determined using the comparative cycle time (ΔΔCt) method.

### Determination of plasma DAO activity by an enzyme-linked immunosorbent assay (ELISA)

Plasma of rats was separated in a refrigerated centrifuge and stored at −20°C before determination of the DAO activity using the rat diamine oxidase ELISA Kit (FineTest; ER0895, Wuhan, China) according to the manufacturer’s instructions. The DAO activity was calculated according to a standard curve and presented in units/L.

### Statistical analyses

At least five rats were used for all experimental groups. The Data of RT-qPCR are represented as mean ± standard error of the mean from three biological replicates for each sample, while the mRNA sample would be collected at least three samples. The percentage contribution of positivity of IHC was calculated for at least 10 positions by image J Software (ImageJ 1.53k, NIH, USA). The data were expressed as the mean ± standard error of the mean (S.E.M). Statistical analyses were carried out with the GraphPad Prism software program (GraphPad Software, Inc., California, USA) using a one-way analysis of variance (ANOVA), t-test, or an analysis of variance with repeated measures when appropriate. The survival rate of the rats was shown with Log-rank (Mantel-Cox) test. Asterisks (*) indicate significant differences (* P < 0.05, ** P < 0.01, by t-test or one-way ANOVA).

## Results

### DKT can enhance the body weight recovery and ameliorate the symptoms of IM induced by MTX

**Fig 1:**
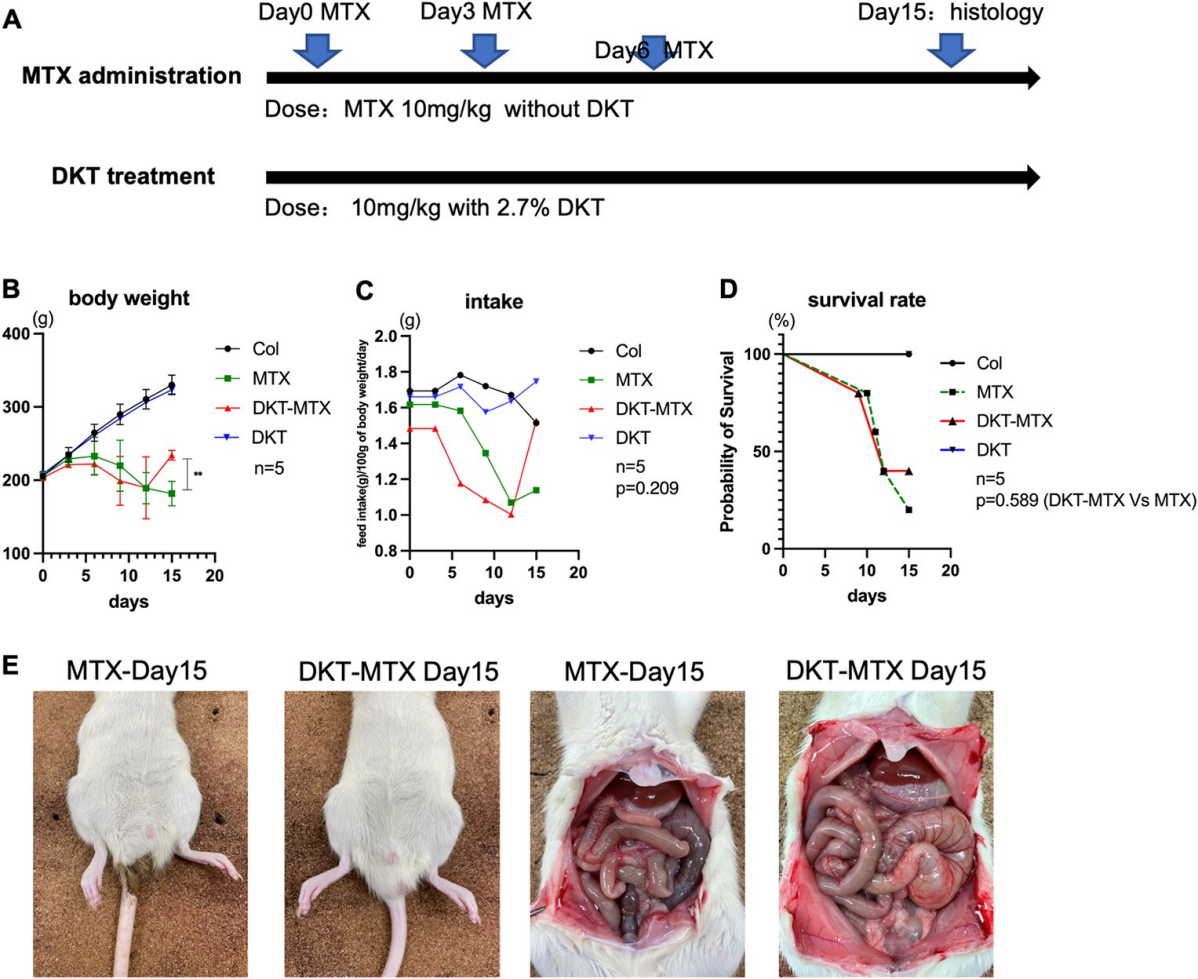
DKT enhanced the body weight recovery and ameliorated the symptoms of IM induced by MTX. **(A)** The experiment was performed according to the diagram. **(B)** The body weight of the rats was shown. Data are represented as mean ± standard error of the mean. One-way ANOWA test, followed by Tukey’s multiple comparison test for day15, ns>0.05, **p < 0.001. **(C)** The food intake of the rats was shown. Data are represented as mean only, Student-t test for day 15 data, DKT-MTX Vs MTX, p = 0.209. **(D)** The survival rate of the rats in all groups was shown. Log-rank (Mantel-Cox) test, p value = 0.5890. **(E)** The rats in the DKT-MTX group had no obvious diarrhea, and the intestinal walls of the rats in the MTX group were thinned.

MTX was used for induction of CIM, and the optimum dose and frequency (10 mg/kg every 3 days, 3 times total) of MTX to induce CIM was determined. The DKT treatment experiment was performed according to the diagram **(Fig 1A)**. According to the lectures and the recommend dose for patients, 2.7% of the total mass mixed in the feed was administered orally from the beginning of the administration of MTX [18]. The body weight of the rats in the MTX and DKT-MTX groups decreased over time, compared with control group, but after day 12, the body weight of the rats in the DKT-MTX group significantly increased **(Fig 1B)**. The changes in food intake were similar to those of the body weight, with rats in both the DKT-MTX and MTX groups showing reductions after the administration of MTX, compared to the control group, although values gradually increased in DKT-MTX group after day 12 **(Fig 1C)**. There was also no significant difference in the survival rate between the MTX group and the DKT-MTX group on day 15 **(Fig 1D)**.

The MTX group had severe diarrhea by day 15, while the rats in the DKT-MTX group had only slightly soft stool. When the small intestine was observed after euthanasia and opening, the small intestine in the MTX group was observed to be relatively pale in appearance **(Fig 1E)**. Pathologic changes in the small intestine would be described in detail below.

### DKT attenuated MTX-induced inflammation and mucosal damage in the small intestinal mucosa

**Fig 2:**
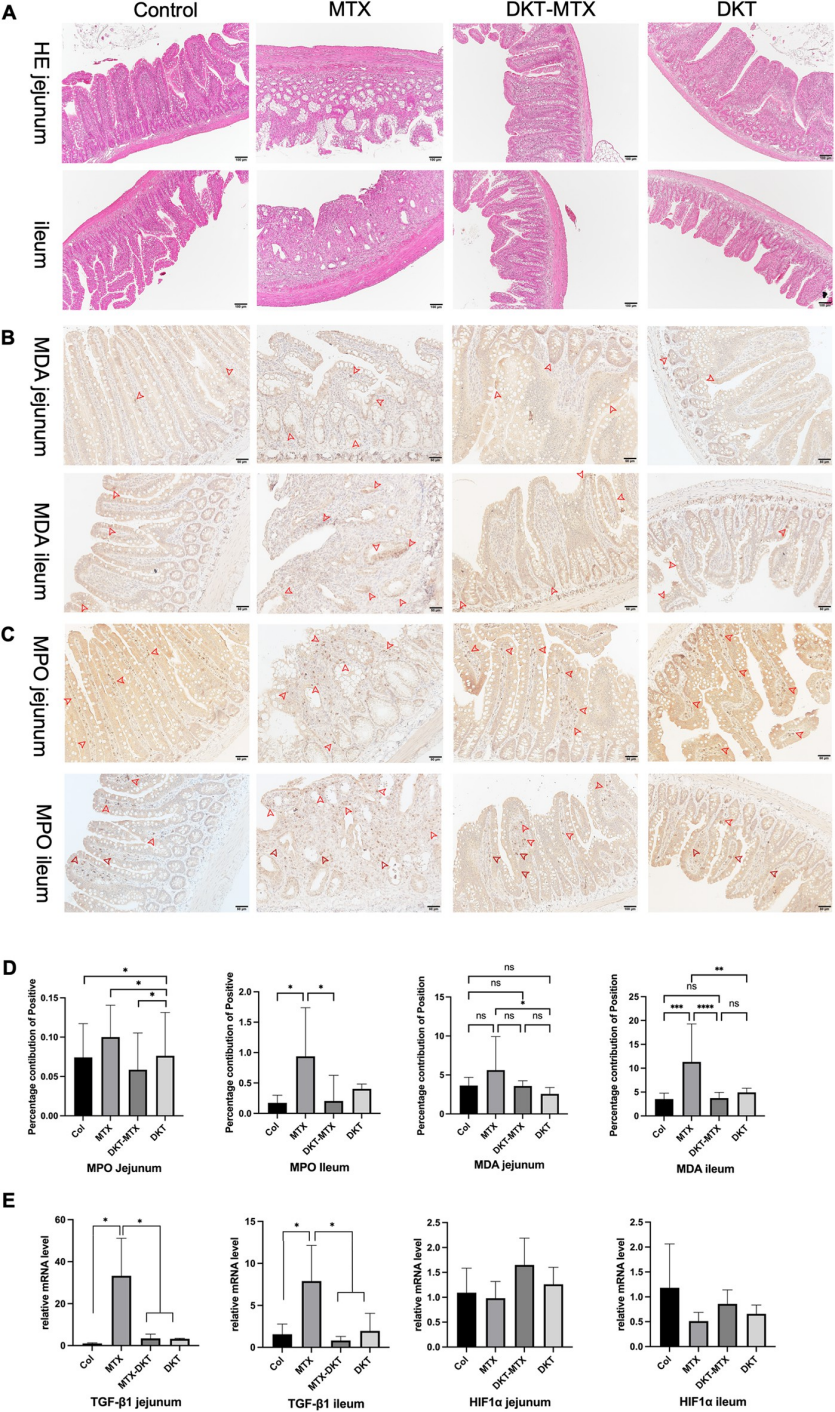
DKT attenuated MTX-induced inflammation and mucosal damage in the small intestinal mucosa. **(A)** HE staining showed the structural changes in all groups. Scale bar = 100 μm. **(B, C, D)** The immunohistochemistry results showed a lower percentage contribution of positivity for **(D)** MDA **(B)** and MPO **(C)** in the jejunum and ileum of the DKT-MTX group than in those of the MTX group. For each criterion, the percentage contribution of positivity was calculated for at least 10 positions. Scale bar = 50 μm. Data are represented as mean ± standard error of the mean. One-way ANOWA test, ns>0.05, *p < 0.05. **(E)** RT-qPCR showed the expression of inflammation-related mRNA of TGF-β1 and HIF-1α in the four groups. Values were determined relative to GAPDH and presented as fold-change relative to the control group. Data are represented as mean ± standard error of the mean from three biological replicates. One-way ANOVA test, ns >0.05, *p <0.05.

Pathological and inflammation-related factors were used to evaluate the inflammation of the small intestinal mucosa and the therapeutic effect of DKT on acute CIM. HE staining showed that the MTX group had degeneration and vacuolization of the surface and crypt epithelium and villus structure in the jejunum, the digestion and dissolution of epithelial structures, and bleeding and edema in the lamina propria in the ileum. The DKT-MTX group showed the degeneration and vacuolization of the surface and crypt epithelium and villus structure in the jejunum and ileum. There were no marked changes in the control or DKT group **(Fig 2A)**.

Immunohistochemical (IHC) staining for MPO and MDA showed that the DKT-MTX group had a lower percentage contribution of positivity than the MTX group in the jejunum and ileum, although there was no significant difference in the MDA in the jejunum **(Fig 2B–2D)**. RT-qPCR showed that the expression of inflammation-related mRNA of TGF-β1 was up-regulated in the small intestine of the MTX group, compared with the DKT-MTX and control groups; however, there were no significant changes in the HIF-1α levels in any groups after DKT treatment **(Fig 2E)**.

### DKT enhanced small intestinal crypt cell proliferation after administration of MTX

**Fig 3:**
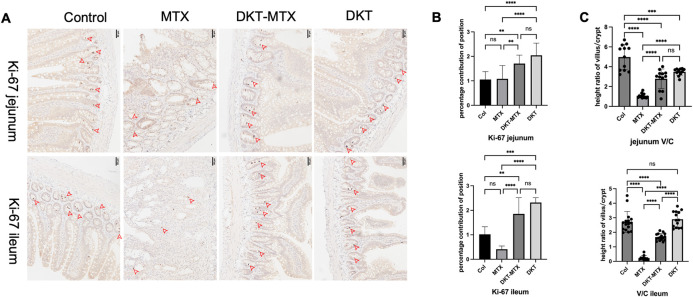
DKT enhanced the small intestinal crypt cell proliferation after the administration of MTX. **(A)** Immunohistochemical results showed that the small intestinal mucosal crypts in the DKT-MTX group contained more Ki-67-positive cells than the MTX group. **(B)** The DKT-MTX group had a higher percentage contribution of Ki-67 positivity than the MTX group. Scale bar = 50 μm. For each criterion, the percentage contribution of Ki-67 positivity was calculated for at least 10 positions using image J software. **(C)** The high ratio of villus to crypt was measured by image J software. Data are represented as mean ± standard error of the mean. One-way ANOVA test, ns >0.05, *p <0.05.

MTX induced small intestinal mucosal epithelial injury by inhibiting the proliferation of small intestinal mucosal crypt cells, which resulted in the suspension of small intestinal mucosal renewal. IHC staining for Ki-67 of the small intestinal tissue was used to evaluate the cell proliferation in the crypts and epithelia of the small intestinal mucosa, which could reflect the repair of the mucosa [19]. After treatment with DKT in the CIM models, the small intestinal mucosal crypts in the DKT-MTX group contained more Ki-67-positive cells than the MTX group **(Fig 3A)**. Positive area counting using the image J software program (NIH, Wisconsin, USA) showed that the DKT-MTX group had a higher percentage contribution of Ki-67 positivity than the MTX group in crypts **(Fig 3B)**. Normal villous crypt ratio (V/C) is 3 to 5:1 [20]. After the rats in the DKT-MTX group were administered with DKT, the small intestinal V/C ratio in the DKT-MTX group was significantly higher than that in the MTX group, which was close to 3 **(Fig 3C)**.

### DKT promoted the repair of the mucosal barrier and nutrient absorption functions

**Fig 4:**
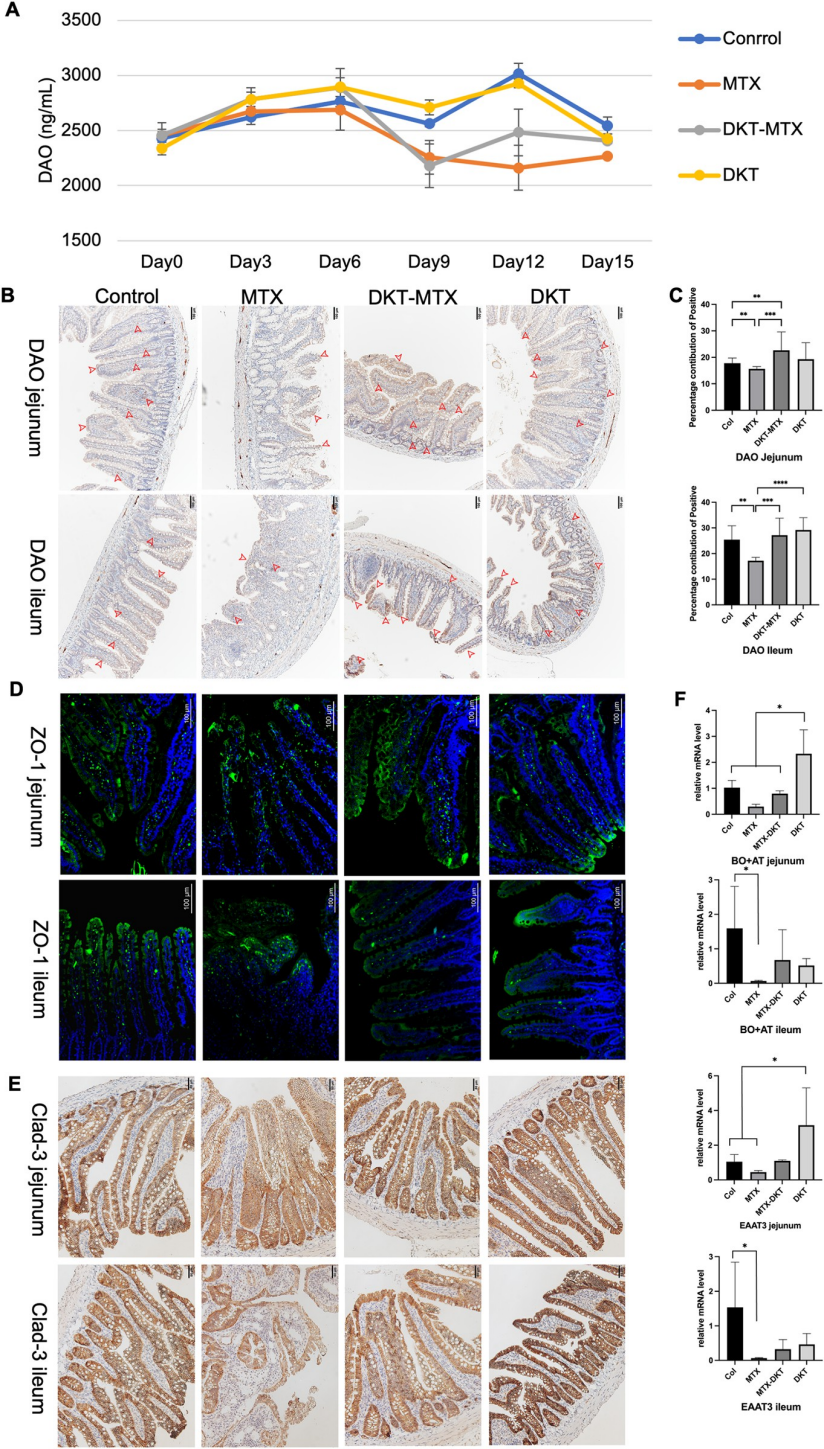
DKT promoted the repair of the mucosal barrier and nutrient absorption functions. **(A)** The plasma DAO level in the control, MTX, DKT-MTX, and DKT groups. The DAO level decreased in both the MTX and DKT-MTX groups compared with the control group but gradually recovered after day 9 in the DKT-MTX group. Units: ng/ml; DAO, diamine oxidase. **(B)** Immunohistochemical staining shows the positions and relative level of DAO in the jejunum and ileum of the control, MTX, DKT-MTX, and DKT groups, and **(C)** the percentage contributions of positivity are shown. Scale bar = 100 μm. For each criterion, the percentage contribution of positivity was calculated for at least 10 positions. Data are represented as mean ± standard error of the mean. One-way ANOVA tests: ns>0.05, *p <0.05. **(D)** Immunofluorescence staining with the anti-Zo-1 antibody showed the expression and positions of the tight junction protein Zo-1. Bar = 100 μm, Zo-1, zonula occludens-1. **(E)** The tight junction related claudin-3 protein associated to the intestinal permeability was investigated by IHC staining. Scale bar = 50 μm **(F)** RT-qPCR showed the relative mRNA levels of BO+AT and EAAT3 in the jejunum and ileum. Values were determined relative to GAPDH and presented as fold-change relative to the control group. Data are represented as mean ± standard error of the mean from three biological replicates. One-way ANOVA: ns>0.05, *p <0.05. EAAT3: Excitatory amino acid transporter 3; B0+AT: Broad neutral amino acid transporter.

DAO can reflect the degree the mucositis induced by MTX. In DKT treatment experiments, the DAO level was decreased in both the MTX and DKT-MTX groups, compared with the control group but gradually recovered after 12 days in the DKT-MTX group **(Fig 4A)**. IHC staining also showed the positions and relative level of DAO in the jejunum and ileum, with statistically significant differences being noted in the jejunum and ileum between the DKT-MTX and MTX groups **(Fig 4B and 4C)**.

To investigate the effect of DKT on the repair of the mucosal barrier function in MTX-induced IM, we examined the ZO-1 protein level in the intestinal tissue by immunofluorescence staining. ZO-1 was predominantly localized along the apical membrane of the intestinal villi in the control, DKT-MTX, and DKT groups. In contrast, a reduction in ZO-1 immunostaining was observed along the apical membrane of the intestinal villi, especially along the ileum, in the MTX group **(Fig 4D)**. Additionally, the tight junction related factor associated to the intestinal permeability, cladin-3, was also investigated. The MTX-treated rats in the MTX group had decreased claudin-3 protein density in the intestinal mucosa, especially in the ileum. The DKT-MTX group had higher claudin-3 protein density in the intestinal mucosa of the rats compared with the MTX group **(Fig 4E)**.

To investigate the effect of DKT on the repair of nutrient absorption function in MTX-induced IM, we examined the expression of genes related to the amino acid transport-related proteins excitatory amino acid transporter 3 (EAAT3) and broad neutral amino acid transporter (B0+AT) in the small intestinal mucosa. The relative gene expression of EAAT3 and B0+AT in the DKT-MTX group was significantly higher than that in the MTX group, especially in the jejunum **(Fig 4F)**.

## Discussion

IM is a common side effect of chemotherapy, and with the increasing number of cancer diagnoses and the prevalence of chemotherapy drug use, chemotherapy-associated mucositis has become increasingly common. MTX, as an anti-cancer drug widely used for leukemia and other malignancies, is a structural analogue of folic acid that can inhibit the metabolism of folic acid by competitively inhibiting dihydrofolate reductase, thereby inhibiting the *de novo* synthesis of purines and pyrimidines [21]. Over the past few decades, MTX has been successfully used alone or in combination with other drugs to treat various cancers and autoimmune diseases [22]. Unfortunately, however, due to its multi-organ toxicity, especially in the gastrointestinal system, the therapeutic potential of MTX can be reduced, with the drug typically having to be ceased in response to bone marrow toxicity, cardiotoxicity, nephrotoxicity, and liver toxicity [23,24]. Damage to the gastrointestinal mucosa following MTX treatment in cancer patients includes villus shortening and fusion, epithelial atrophy, crypt loss, inflammatory infiltration of the lamina propria, goblet cell depletion, and barrier dysfunction due to loss of mucosal integrity and reduced nutrient absorption [25].

MTX-induced IM not only causes direct damage by DNA copy inhibition but also induces inflammation and the generation of reactive oxygen species (ROS) [26]. A study has demonstrated that MPO and MDA levels are increased after treatment with MTX, suggesting the possible participation of neutrophil infiltration and ROS in MTX-induced IM [26]. In the present study, we found that levels of MPO, a marker of neutrophil accumulation and infiltration during intestinal mucosa damage, were increased in the epithelial mucosa of rats with IM induced by MTX. Kolli et al. also reported that oxidants such as MDA, which is a product of lipid peroxidation, were increased in MTX-induced small IM [27]. MPO is also an enzyme with peroxidative damage secreted by neutrophils after neutrophil, and it also causes peroxidative damage to tissues. According to our results, oral administration of DKT can inhibit the inflammatory process and ROS damage, as evidenced by the fact that DKT can reduce the production of MPO and MDA.

In addition to peroxidation-related factors, growth factors secreted from mucosa also played a role in this process. TGF-β1 and HIF-1α were reported to protect intestinal integrity [28,29]. The mRNA level of TGF-β1 in the DKT-MTX group was higher than that in the control group on day 15, but it was lower in the jejunum and ileum than in the MTX group. This method of mucosal repair was also verified in another experiment of DKT in the treatment of intestinal injury repair in rats [12].

The changes in the level and localization of ZO-1 induced by MTX may lead to disorder of the barrier function, which leads to increased intestinal permeability, resulting in intestinal mucosal barrier dysfunction [30]. ZO-1, as a tight junction scaffold protein, has been shown to render structure firmness and impermeability to the junction and functions as a link between occludin and actin, which are the major elements in the structure of the barrier of the small intestine [31]. However, Kuo et al. reported that the tight junction protein ZO-1 was dispensable for the barrier function but critical for intestinal mucosal repair [32]. Furthermore, redistribution of this tight junction function along the lateral plasma membrane sustained the epithelial barrier during cell shedding [33]. In the present study, an immunohistochemical analysis revealed that ZO-1 was predominantly localized along the apical membrane of the intestinal villi in the control and DKT groups. DKT has the potential to promote the expression of ZO-1 in the small intestinal mucosa epithelium to facilitate the repair of tight junctions and improve the mucosal nutrient absorption. These results were also confirmed by the immunostaining of the claudin-3.

The expression of genes related to the amino acid transport-related proteins EAAT3 and B0+AT was up-regulated after the administration of DKT in the DKT-MTX group. A prospective study of the effects of DKT on the blood flow in the superior mesenteric artery and portal vein (PV) reported that DKT may modulate the SMA and PV blood flows by acting on intestinal micro-vessels [34]. The absorption of nutrients around the ileocecal region was related to the blood flow of the intestine and PV. A prospective open-labeled randomized exploratory study also demonstrated that DKT can improve the perioperative nutritional status of patients with colorectal cancer [35].

DAO is a highly active intracellular enzyme in the upper villi of the human and mammalian small intestinal mucosa that plays a role in the metabolism of histamine and various polyamines [36]. Plasma DAO activity was reported to be associated with the degree of small intestinal injury and had potential utility for measuring mucositis during chemotherapy [36,37]. A previous study showed that an increase of DAO activity in the intestine of mature rats leads to an increase in the degree of DAO activity in plasma [38]. In contrast, intestinal mucosal damage caused by hypertonic sodium sulfate solution or atrophy caused by a low-fiber diet reduces plasma DAO activity [39]. In the present study, with the occurrence of MTX-induced IM, the DAO content in rat plasma decreased, and with the onset of gastrointestinal tract symptoms aggravated and reduced to a lower level. In the DKT-MTX group, as DKT promoted the recovery of rat mucosa, the serum content of DAO was also increased. The IHC results also confirmed that the rat small intestinal mucosal epithelial villi of the DKT-MTX group contained higher DAO levels in the mature apical membrane than that in MTX group.

DKT is mainly composed of dried Japanese pepper extract, ginger extract, and ginseng extract. Ginger can reportedly improve ileum damage caused by MTX, shortened villus fusion, inflammatory cell infiltration, and goblet cell depletion [40]. As one of the main components of DKT, ginger extract has been verified to have a variety of intestinal effects, including anti-inflammatory and antioxidative effects in ulcerative colitis [41]. The mechanism underlying the restoration of the intestinal barrier function by ginger extract involves the increased expression of ZO-1 and claudin-1 protein [42]. Regarding dried Japanese pepper extract (*Zanthoxylum fructus*), there have been articles reporting that *Zanthoxylum fructus* extract inhibits the reduction in mast cell activation by inhibiting sphingosine kinase 1, mainly reducing the release of inflammatory mediators [43]. In research on DKT, it is reported that DKT depolarizes the pacemaker potential of Cajal interstitial cells in an internal or external Ca^2+^-dependent manner by stimulating the 5-HT4 and muscarinic M3 receptors. Its main ingredients are ginseng and ginger root, which help DKT regulate the activity of the intestine and reduce the dysfunction of the small intestine [44]. Some researchers have screened cytoprotective agents against MTX-induced cell genotoxicity from among biologically active phytochemicals and found that agents, such as Siberian ginseng and curcumin have cytoprotective effects [45]. Total ginsenosides was reported to promote intestinal epithelial cell proliferation, presumably via the regulation of the cell cycle and of the expression of proliferation-related proteins by polyamines [46]. Specifically, DKT appears to be less effective in reducing rat mortality due to severe mucosal necrosis but shows marked efficacy in another aspect, such as reducing injury and promoting mucosal repair, as well as improving symptoms. Furthermore, DKT may also have some efficacy against chronic mucositis of the small intestine, so a further investigation regarding chronic mucositis in the clinical setting or as a daily disease will be needed in the future, with particular focus on the therapeutic effect of DKT on chronic mucositis of the small intestine.

In conclusion, DKT may be able to protect against MTX-induced acute small intestinal mucosal injury in a rat model via anti-peroxidation, stimulating cell proliferation, and stabilizing the mucosal barrier. Although DKT comprise numerous chemically diverse compounds with multi-target effects, it has been shown to be able to protect and treat intestinal injury, regardless of administration as a single component or a mixture of multiple components.

## Supporting information

S1 Table List of the first and secondary antibodies in the experiments. (DOCX)

S2 Table List of the Taq-man primer of Rt-PCR performed in the experiments. (DOCX)
