# Supplementary material for: Therapeutic effect and mechanism of Daikenchuto in a model of methotrexate-induced acute small intestinal mucositis
Source: PLoS One. 2023 Mar 30;18(3):e0283626. doi: 10.1371/journal.pone.0283626 (PMC10062645; doi:10.1371/journal.pone.0283626)
Supplement: S1 Table — (DOCX) [file pone.0283626.s001.docx]

**S1 Table. List of the first and secondary antibodies in the experiments.**

| Ki-67 antibody | Ab16667 | Abcam |
| --- | --- | --- |
| MDA antibody | Ab6463 | Abcam |
| TGF-beta1 antibody | Ab215715 | Abcam |
| MPO antibody | ab9535 | Abcam |
| Rat diamine oxidase (DAO) antibody | MBS2025695 | MYBiosource,Inc |
| Rat diamine oxidase (DAO) ELISA Kit | ER0895 | Wuhan Fine Biotech Co., Ltd. |
| Zo-1 antibody | ab10085 | Abcam |
| Claudin-3 | ab15102 | Abcam |
| Anti-rabbit IgG-peroxidase | A0545 | Sigma-Aldrich Co. LLC. |
| Anti-goat IgG-peroxidase | A5420 | Sigma-Aldrich Co. LLC. |
| Anti-mouse IgG-peroxidase | A9044 | Sigma-Aldrich Co. LLC. |
