# Supplementary material for: Therapeutic effect and mechanism of Daikenchuto in a model of methotrexate-induced acute small intestinal mucositis
Source: PLoS One. 2023 Mar 30;18(3):e0283626. doi: 10.1371/journal.pone.0283626 (PMC10062645; doi:10.1371/journal.pone.0283626)
Supplement: S2 Table — (DOCX) [file pone.0283626.s002.docx]

**S2 Table: List of the Taq-man primer of Rt-PCR performed in the experiments.**

| Gene Aliases | Gene symbol | Gene name | TaqMan® ID | Amplicon Length | Target species |
| --- | --- | --- | --- | --- | --- |
| Gapdh | Gapdh | Glyceraldehyde-3-phosphate-dehydrogenase | Rn99999916_s1 | 87 | Rat |
| Tgf-β1 | Tgf-β1 | Transforming growth factor beta 1 | Rn00572010_m1 | 65 | Rat |
| Hif-1α | Hif-1α | Hypoxia inducible factor 1, alpha | Rn01472831_m1 | 90 | Rat |
| BO+AT | Slc7a9 | Solute carrier family 7 (Amino Acid Transporter Light Chain, Bo, + System) | Rn00588400_m1 | 73 | Rat |
| Eaat3 | Slc1a1 | Broad neutral amino acid transporter | Rn00564705_m1 | 92 | Rat |
| Zo-1 | Tjp1 | tight junction protein 1 | Rn07315717_m1 | 64 | Rat |
